# Metastasis to the Thyroid Gland: A Critical Review

**DOI:** 10.1245/s10434-016-5683-4

**Published:** 2016-11-21

**Authors:** Iain J. Nixon, Andrés Coca-Pelaz, Anna I. Kaleva, Asterios Triantafyllou, Peter Angelos, Randall P. Owen, Alessandra Rinaldo, Ashok R. Shaha, Carl E. Silver, Alfio Ferlito

**Affiliations:** 1 ENT Department, NHS Lothian, Lauriston Building, Lauriston Place, Edinburgh, UK; 2 Department of Otolaryngology, Hospital Universitario Central de Asturias, Oviedo, Spain; 3 ENT Department, East and North Hertfordshire Trust, Stevenage, UK; 4 Oral and Maxillofacial Pathology, School of Dentistry, University of Liverpool, Liverpool, UK; 5 Department of Cellular Pathology, Liverpool Clinical Laboratories, Liverpool, UK; 6 Department of Surgery and MacLean Center for Clinical Ethics, The University of Chicago Medicine, Chicago, IL USA; 7 Division of Surgical Oncology, Department of Surgery, Mount Sinai School of Medicine, New York, NY USA; 8 University of Udine, Udine, Italy; 9 Head and Neck Surgery, Memorial Sloan Kettering Cancer Center, New York, NY USA; 10 Department of Surgery, University of Arizona College of Medicine—Phoenix, Phoenix, AZ USA; 11 International Head and Neck Scientific Group, Padua, Italy

## Abstract

**Background:**

Metastasis to the thyroid gland from nonthyroid sites is an uncommon clinical presentation in surgical practice. The aim of this review was to assess its incidence management and outcomes.

**Methods:**

A literature review was performed to identify reports of metastases to the thyroid gland. Both clinical and autopsy series were included.

**Results:**

Metastases to the gland may be discovered at the time of diagnosis of the primary tumor, after preoperative investigation of a neck mass, or on histologic examination of a thyroidectomy specimen. The most common primary tumors in autopsy studies are from the lung. In clinical series, renal cell carcinoma is most common. For patients with widespread metastases in the setting of an aggressive malignancy, surgery is rarely indicated. However, when patients present with an isolated metastasis diagnosed during follow-up of indolent disease, surgery may achieve control of the central neck and even long-term cure. Other prognosticators include features of the primary tumor, time interval between initial diagnosis and metastasis, and extrathyroid extent of disease.

**Conclusions:**

In patients with thyroid metastases, communication among clinicians treating the thyroid and the index primary tumor is essential. The setting is complex, and decisions must be made considering the features of the primary tumor, overall burden of metastases, and comorbidities. Careful balancing of these factors influences individualized approaches.

The earliest description of metastasis to thyroid gland is attributed to Virchow, who described a testicular tumor metastasis in 1871. Given the extensive blood supply to the gland, the low incidence of metastases to the thyroid is surprising^1^. Willis suggested that this is influenced by the glandular microenvironment; the fast arterial blood flow and high concentration of oxygen and iodine could thus prevent the anchorage and secondary growth of circulating tumor cells.^1^

While postmortem examination suggests that as many as 24% of patients who die of nonthyroid malignancies have metastases to this gland, these seem rare in clinical practice.^2^–^4^ Although the majority (60–80%) of patients who present with thyroid metastasis are diagnosed in the setting of known previous malignancy, an occult primary tumor accounts for a significant percentage (20–40%).^4^,^5^ In addition, some patients are diagnosed during preoperative investigation, while others will be diagnosed on histologic examination of a thyroidectomy specimen.

## Primary Origin of Thyroid Metastases

**Fig. 1:**
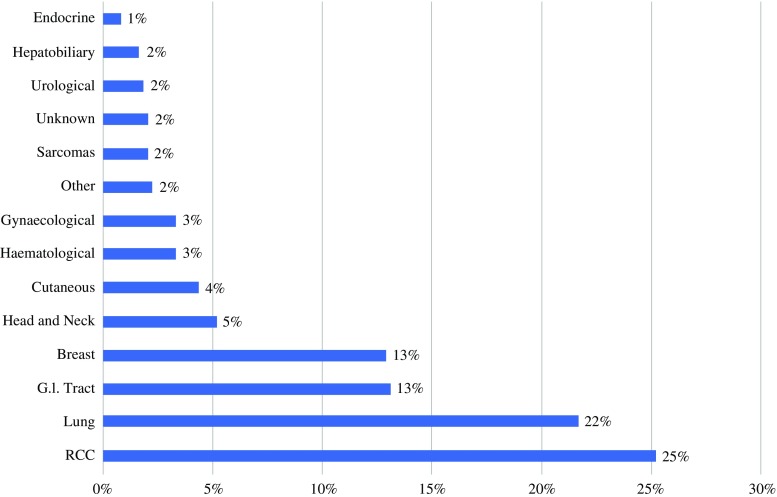
Total number of cases of metastasis to thyroid reported in literature stratified by site of primary tumor

In autopsy series, the incidence of metastases overall is 2%. In autopsy series, the most common site of primary tumor is the lung, whereas in clinical series renal cell carcinoma (RCC) is more common (Table 1).^1^,^6^–^18^ Because of the aggressive nature of lung malignancies, patients are often treated with palliative intent from an early stage, and investigation for additional metastases is therefore curtailed. In contrast, RCC is less aggressive, and patients are more likely to be further investigated and treated for metastatic disease. Metastasis to the thyroid should not be confused with direct infiltration of the thyroid by locally aggressive disease in the larynx or upper esophagus, as reported in some series.^19^ Metastases can originate at almost any primary site^20^–^27^ Figure 1 summarizes the most common primary tumors reported in clinical series to metastasize to the thyroid (Table 2).^21^,^26^,^28^–^51^

**Table 1:** Frequency of metastasis to the thyroid gland in autopsy series

Study	No. thyroid metastases/no. of autopsies	%	Most frequent site of primary tumor
Müller^8^	11/623	1.8	Not available
Symmers^9^	7/298	2.3	Not available
Kitain^10^	14/452	3.1	Not available
Willis^1^	9/170	5.2	Breast
Rice^11^	9/89	10.1	Not available
Abrahms et al.^12^	19/1000	1.9	Breast
Thorpe^13^	4/200	2	Not available
Hull^14^	10/59	16.9	Lymphoid tissue
Mortensen et al.^15^	18/467	3.9	Lymphoid tissue
Shimaoka et al.^7^	188/1980	9.5	Breast
Brierre and Dickinson^16^	14/53	26.4	Not available
Silverberg and Vidone^77^	15/62	24.2	Lung
Berge and Lundberg^51^	201/16294	1.25	Breast
Watanabe and Tsuchiya^18^	4/309	1.29	Lung
Moghaddam et al.^6^	4/2117	0.46	Lung
Total (mean)	527/24173	2	Lung

**Table 2:** Incidence of primary tumors metastasising to the thyroid and treatment as reported in clinical series

Study	*n*	Most frequent site of primary tumor	Total thyroidectomy (%)
Mayo et al.^42^	19	Lung	Not available
Elliot et al.^34^	14	Breast = lung	43
Wychulis et al.^43^	14	Kidney	14
Harcourt-Webster^35^	11	Skin	0
Pillay et al.^44^	10	Esophagus	10
Brady et al.^36^	14	Lung	Not available
Ericsson et al.^33^	10	Kidney	70
Czech et al.^32^	12	Kidney	100
Ivy et al.^37^	30	Kidney	Not available
Chacho et al.^45^	8	Blood	0
Smith et al.^46^	19	Breast	0
Michelow et al.^47^	21	Lung	0
Rosen et al.^21^	11	Lung	55
Nakhjavani et al.^28^	43	Kidney	21
Lam et al.^38^	79	Lung	Not available
Chen et al.^40^	10	Kidney	50
De Ridder et al.^26^	6	Kidney	67
Dequanter et al.^48^	11	Lung	82
Wood et al.^30^	15	Kidney	47
Kim et al.^29^	22	Breast	9
Mirallié et al.^49^	29	Kidney	72
Cichoń et al.^31^	17	Kidney	94
Gerges et al.^39^	7	Lung	0
Choi et al.^50^	46	Lung	0
Russell et al.^41^	17	Kidney	65

## Preoperative Investigation

An accurate clinical history is significant. A prior malignancy, as well as symptoms such as hematuria or hemoptysis, may raise the possibility of an occult primary tumor in the kidneys or lungs. However, many patients with thyroid metastasis present with signs and symptoms identical to those with primary thyroid disease. One study indicates that 72% present with a palpable neck mass and 28% with an incidental lesion identified on imaging.^52^ Thyroid metastasis at an advanced stage within the central neck may result in dysphagia and dysphonia, similar to aggressive thyroid malignancy. Changes in thyroid function are late and relatively uncommon.^5^,^53^

The accuracy of thyroid imaging has improved with the introduction of high-resolution ultrasound as well as cross-sectional [computed tomography/magnetic resonance imaging (CT/MRI)] and functional imaging [positron emission tomography (PET)]. Nevertheless, even sophisticated techniques cannot reliably differentiate between primary thyroid lesions and metastases.^28^,^54^ Fine needle aspiration cytology (FNAC) can often be of assistance.^55^,^56^ In the setting of metastatic lesions to the thyroid, FNAC positive and negative predictive values of 89 and 93%, respectively, have been reported, and application of molecular markers (e.g., the *BRAF* growth promoter in papillary thyroid cancer) and immunohistochemistry may be of further help (e.g., CD-10 in RCC).^25^,^57^–^59^ There limitations of cytology are acknowledged.^29^,^56^,^60^ Poorly differentiated tumors such as aggressive anaplastic thyroid cancers can be difficult to differentiate from-high grade metastases.^54^,^58^,^61^,^62^ In addition, neck hematoma after needle biopsy of RCC has anecdotally been seen and should raise concerns for performing core biopsies of lesions suspicious for RCC. Rare, and hence morphologically unfamiliar, tumors and lack of cellular differentiation and expression of antigens conveniently detected by immunohistochemistry account for some of the difficulties.^2^,^61^ To increase the likelihood of a preoperative diagnosis, more invasive investigations such as core or open biopsy have been considered.^4^,^30^ Obviously, the multidisciplinary team must have a high index of suspicion, particularly in patients with a history of malignancy.

## Aims of Treatment

Many patients who present with a thyroid metastasis will be treated with palliative intent. For selected patients, however, lobectomy or total thyroidectomy may be performed, either with the aim of long-term cure or achieving local control.^31^ A balance must be reached regarding the course of systemic disease versus the likely outcome from uncontrolled local disease. Because of the nature of reported surgical series, the percentage of patients who present with metastasis to the thyroid who are considered candidates for surgery is unclear. Local invasion of thyroid disease, irrespective of the source of malignancy, results in dysphonia, dysphagia, hemoptysis, and stridor. Unfortunately, few data exist on the frequency of presenting symptoms. In addition, the patient’s fitness and comorbidities need to be weighed against the likelihood of surgical success. The necessary close communication between the teams involved in the management of primary disease and thyroid is ensured by modern multidisciplinary team management.

If the metastasis is confined within the thyroid gland without evidence of significant extraglandular extension, thyroidectomy may be performed with minimal morbidity. In appropriately selected cases, the aim would be to prevent asphyxia and hemoptysis associated with uncontrolled disease in the central neck.^32^,^33^

In the case of a relatively indolent primary malignancy present with an isolated thyroid metastasis presenting many years after treatment for the index tumor, surgery with curative intent is possible. Continuous communication between the management teams positively influences patient counseling and treatment planning.

## Prognostic Factors

Although distant metastases are often an adverse prognosticator, thyroid metastases may not have as poor an outcome as those elsewhere.^52^ Nevertheless, 35 to 80% of patients with thyroid involvement present with multiorgan metastases.^5^,^52^,^63^

Overall prognosis appears to be most closely linked to the innate features of the primary tumor.^5^ As already noted, if the primary tumor is amenable to treatment with curative intent, the subgroup of patients with isolated thyroid metastasis would be candidates for curative treatment of the metastasis as well.^19^,^64^ When considering thyroid surgery in a palliative setting, the thyroid team should consider the burden/volume of metastatic disease elsewhere and the effect of extrathyroidal extension on surgical morbidity, but individualized decisions are complex.^65^

For those patients with an indolent primary lesion and disease confined to the thyroid gland without significant extrathyroid extension, outcomes may be favorable. In contrast, patients with aggressive primary disease, multiorgan metastases, and invasive thyroid metastases will fare less well.

Mean survival after surgery for thyroid metastasis is approximately 2 years, with 42% 5-year overall survival.^4^,^63^ However, in the majority of those patients who are selected for thyroid metastasectomy, long-term control of the central neck can be achieved.

In the case of RCC, approximately 20% of these patients are diagnosed with distant metastases at the time of diagnosis. Another 30% will go on to develop metastases during follow-up, and some of these may present after a significant delay of up to 20 years.^66^–^68^ Overall prognosis for these patients is poor, but thyroid metastasectomy for selected patients may offer good survival rates (30–50%), and long disease-free intervals are reported.^69^ Hence, the European Association of Urology guidelines support treatment of thyroid metastases with surgery.^70^

Of the lung tumors known to metastasize to the thyroid, non–small cell lung cancer is the most common type.^71^ With regard to breast cancer, 5 to 10% of cases present with distant metastases at the time of diagnosis, and occasionally these are in the thyroid.^72^,^73^ Evidence relating to the management of thyroid metastases in cases of lung and breast cancer is limited, though outcomes appear poor.^6^,^19^,^21^,^28^,^30^–^32^,^34^–^39^,^65^

Colorectal cancer has also been reported to be associated with a low incidence of thyroid metastases. One meta-analysis identified 31 cases of thyroid metastases reported between 1954 and 2006.^74^ These were usually accompanied with multiorgan distant metastases, and hence, prognosis was poor, with only a 50% survival at 1 year.^74^ Treatment for these patients was most commonly thyroidectomy with adjuvant chemotherapy, radiotherapy, or both.^60^ The limited evidence available suggests that despite being palliative, thyroidectomy in this setting was associated with reduced morbidity from thyroid-associated respiratory symptoms.^74^ Melanoma has been associated with thyroid metastases, and autopsy studies report an incidence of up to 39%.^5^,^7^ Shimaoka et al. analyzed 2050 consecutive autopsy reports between 1955 and 1960. Sixteen (39%) of 41 patients who died of malignant melanoma had metastatic deposits in the thyroid gland.^7^ It is possible that as therapy has improved for this disease, rates may currently be lower. As is the case for other primary lesions, the literature mainly consists of case reports and case series. Hence the management and likelihood of success are undetermined. Additional case reports include small cell lung cancers, pancreatic malignancies, and sarcomas.^21^,^28^,^39^,^40^,^71^ The majority of these presented with multiple metastases against a background of aggressive disease, and as such are inappropriate for thyroid surgery.

In summary, the literature lacks prospective randomized trial evidence, and this is unlikely to ever be available. Prognosis largely depends on the features of the primary malignancy and the metastatic profile at the time of diagnosis—that is, timing, number, and location of metastases.

## Surgical Strategy

A recent meta-analysis has suggested that those patients managed with surgery experience better outcomes than those managed expectantly.^41^ This was most apparent for RCC, where median survival for those managed expectantly was 6 months versus 27 months for those who underwent surgery. However, it is likely that studies included in this were subject to significant bias, with an expectant approach favored in patients with more aggressive disease.

For those patients considered to be candidates for surgery, when considering the extent of thyroidectomy, the aim should be to ensure removal of all gross disease with an adequate margin. The procedure will therefore depend on the extent of disease. In unilateral disease, most authors recommend thyroid lobectomy rather than total thyroidectomy in order to minimize risk to the contralateral recurrent laryngeal nerve and parathyroid glands (Table 2). However, some authors suggest that lobectomy can be associated with positive margins and therefore favor total thyroidectomy.^65^ Russell et al. recently demonstrated a decrease in recurrence for patients managed with total thyroidectomy versus thyroid lobectomy (13 vs. 5%, *p* < 0.005), although studies included in this meta-analysis are likely to have been subject to some selection bias.^41^ In contrast to primary thyroid malignancy, metastases to the gland are not sensitive to radioactive iodine; therefore, total thyroidectomy is not mandatory as long as adequate margins are achieved. However, patients with multifocal disease may require primary total thyroidectomy^3^,^5^ When Russell et al. reported their own institutional experience of 17 patients with metastases to the thyroid, 4 (24%) had bilateral disease.^41^ In the rare instance that multifocal disease is identified after initial thyroid lobectomy, consideration may be given to completion thyroidectomy.

Concomitant regional lymph node involvement is rare in cases of metastasis to the thyroid; therefore, prophylactic neck dissection is not recommended.^58^,^60^,^65^,^75^ However, the regional lymphatics should be fully assessed preoperatively, particularly in RCC.^68^ RCC shows a tendency toward vascular invasion, and involvement of the internal jugular vein has been described both from nodal metastases to the lymph nodes of the neck and from metastases to the thyroid gland.^65^ For this reason, contrast-enhanced imaging of the large vessels of the neck should be considered to assess the relationship between disease and vasculature before surgery.

Currently there is no evidence to support any other adjuvant or alternative treatment to surgery.^65^ This is partly because the most common primary tumor is RCC, which is largely considered to be resistant to radiotherapy.^76^

## Summary

Autopsy and clinical series provide relevant data. Nonthyroid metastases to the thyroid gland are rare in the clinical setting, with the most common primary tumor being RCC. In autopsy series, the most common primary tumor is lung cancer. Thyroid involvement may be identified during initial staging, at follow-up imaging, or as a new presentation of a neck mass. Preoperative evaluation is similar to that for thyroid primary disease. Awareness of previous malignancy is of significance and would be particularly helpful while assessing FNAC or core/open biopsies. Prognosis will depend on the biology of the primary tumor, the burden/volume of metastases, the course of disease, and any comorbidities. Communication between oncologic teams is essential and influences decision making. In patients selected for surgery, thyroid lobectomy with adequate margins may achieve control of the central neck, whereas total thyroidectomy should be reserved for large-volume or multifocal disease.
